# A comprehensive review of diagnostic approaches for hepatitis D

**DOI:** 10.3389/fmolb.2025.1598784

**Published:** 2025-05-21

**Authors:** Wen-Hui Liu, Jia-Yue Cui, Miao Yu, Xiao-Dong Shi, Yi-Lin Che, Chu-Yan Wang, Xiu-Mei Chi

**Affiliations:** 1 Core Facility of the First Hospital of Jilin University, Changchun, China; 2 Department of Histology and Embryology, College of Basic Medical Sciences, Jilin University, Changchun, China; 3 Department of Rheumatology and Immunology, First Hospital of Jilin University, Changchun, China; 4 School of Public Health Jilin University, Changchun, China

**Keywords:** hepatitis D, HDV, HDV RNA, HDAg, anti-HDV, diagnosis, screening

## Abstract

Current estimates suggest 9 million to 19 million people worldwide are affected by Hepatitis D virus (HDV) infection, though significant discrepancies in diagnostic guideline implementation across regions and countries indicate these figures may not fully capture the true disease burden. HDV coinfection and superinfection with hepatitis B hasten disease progression, increasing cirrhosis and liver cancer risks, highlighting the importance of early and precise diagnosis. We present a thorough analysis of current and emerging hepatitis D diagnostic methods. Initial diagnosis involves detecting serum anti-HDV antibodies using radioisotope- or enzyme-linked immunosorbent assays. Established techniques like chemiluminescence immunoassay, quantitative microarray antibody capture, and lateral flow assays are being improved. Additional diagnostic markers include HDV antigens and RNA in the serum or liver, detectable through methods like northern and slot blots, fluorescence *in situ* hybridization, and quantitative real-time PCR. Droplet digital PCR allows quantifying unedited and edited HDV genomes in one sample. Next-generation sequencing offers deeper insights into HDV quasispecies for precise genotyping. Challenges persist, including qualitative diagnostic methods and need for international standards due to lab variability. This review emphasizes the urgency of establishing standardized protocols and international standards for early interventions and reducing the medical burden of chronic HDV infection.

## 1 Introduction

**FIGURE 1:**
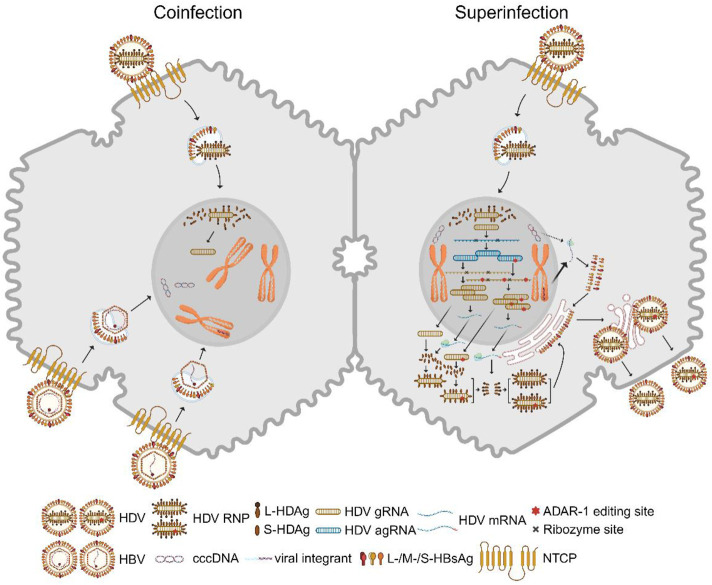
HDV replication in hepatocytes. Left: A hepatocyte is co-infected with HBV and HDV. Both viruses bind to the entry receptor NTCP. The intracellular HDV RNP complex is transported into the nucleus. After membrane fusion, cytoplasmic HBV nucleocapsids shuttle to the nucleus, where rcDNA is converted to cccDNA and double-stranded linear DNA integrates into host chromosomes. Right: HDV superinfection occurs with a single HDV infection in the hepatocyte where cccDNA and HBV viral integrants stably exist. HBV envelope proteins are predominantly expressed from the integrants rather than cccDNA in this case. Nuclear HDV gRNA with negative polarity is the template for a first rolling circle amplification, which generates agRNA dimers and trimers. These multimers are self-cleaved by ribozyme activity in cis and ligated into covalently closed circular agRNA. A portion of agRNA is edited by ADAR1, which alters an amber codon to tryptophan and thereby extends the HDAg coding region. Unedited and edited agRNA undergo a second rolling circle amplification that generates gRNA multimers and monomers. Circular gRNAs are the template for mRNA transcription which is translated into S-HDAg and L-HDAg. Both viral antigens bind gRNA and form RNPs which are exported into the cytosol. L-HDAg is responsible for the interaction between RNP and HBV envelope proteins but is also prenylated at the C-terminal four amino acids by farnesyltransferase which is required for envelopment. HDV virions are presumably released via the classic secretory pathway. ADAR1: adenosine deaminases acting on RNA 1; agRNA: antigenomic RNA; gRNA: genomic RNA; cccDNA: covalently closed circular DNA; rcDNA: relaxed circular DNA; NTCP: sodium taurocholate cotransporting polypeptide; RNP: ribonucleoprotein. S/L-HDAg: small/large HDV antigen. Created with BioRender.com.

In 1977, Rizzetto and colleagues identified a novel viral antigen-antibody system in patients with hepatitis B virus (HBV) infection, which they named hepatitis D virus (HDV) (Rizzetto et al., 1977; Rizzetto et al., 1980). HDV, belonging to the *Deltavirus* genus, is a small defective RNA virus, with a genome length ranging from 1,672 to 1,697 base pairs (Lai, 1995). HDV depends on the presence of HBV surface antigens (HBsAg) to form infectious virions and enter hepatocytes (Rizzetto et al., 2021; Yan et al., 2012). HDV’s genome, a negative-stranded circular RNA (gRNA), encodes two specific HDV antigens (HDAg): a 24kD small (S)-HDAg and a 27kD large (L)-HDAg, which play crucial roles in HDV RNA replication and virion assembly. The HDV gRNA undergoes initial rolling circle amplification, producing positive-stranded antigenome (agRNA), which can be edited by adenosine deaminase ADAR1. Both unedited and edited agRNAs are converted back to gRNAs through a second amplification step and serve as templates for mRNA translation, yielding S-HDAg and L-HDAg (Figure 1) (Lucifora and Delphin, 2020).

Like HBV, HDV is primarily transmitted through the exchange of blood and bodily fluids, including vertical transmission, intravenous drug use, and risky sexual behaviors, particularly in areas with poor sanitation (Chen et al., 2019; Niro et al., 1999). Meta-analyses indicate varying numbers of hepatitis D cases worldwide, with estimates ranging from 12 to 72 million, and the prevalence of anti-HDV antibodies (anti-HDV) among HBsAg-positive populations being approximately 4.5%–13.0% (Chen et al., 2019; Stockdale et al., 2020; Miao et al., 2020). Current global estimates are subject to ongoing debate, as surveillance gaps in regions with limited HDV testing capacity coexist with methodological inconsistencies in clinical data collection arising from non-standardized diagnostic practices. Chen et al. found that HDV patient’s rates were highest in Africa, the Amazon Basin, Eastern Europe, parts of the Mediterranean, the Middle East, and Asia (Chen et al., 2019). Furthermore, there are eight HDV genotypes, with HDV-1 being globally prevalent, while other genotypes exhibit geographical variations (Le Gal et al., 2017). The percent similarity between HDV genotypes can be as low as 64%, leading to variations in viral markers, antibody responses, and diagnostic applications (Le Gal et al., 2006).

Compared to HBV mono-infection, HDV superinfection accelerates the progression to chronicity, increasing the risk of cirrhosis, liver cancer, and other complications (Rizzetto et al., 2021). Therefore, chronic HDV infection is considered the most severe form of viral hepatitis. Given that more than 12 million people test positive for anti-HDV antibodies, and the actual number of infections may be underestimated, there is an urgent need for a specific and sensitive diagnostic method. This review provides a summary and comparison of current and innovative approaches for diagnosing hepatitis D.

## 2 Viral replication and antibody responses during HBV-HDV coinfection and HDV superinfection

**FIGURE 2:**
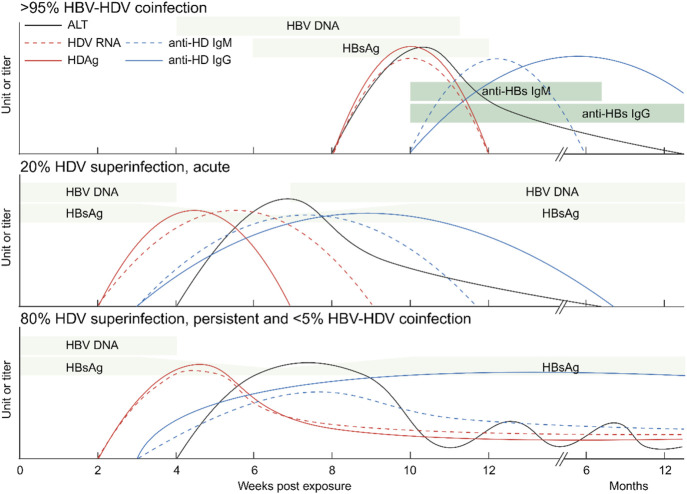
Serological markers of hepatitis D. Levels of HDV RNA, HDAg, anti-HDV IgM and anti-HDV IgG as well as ALT are shown. Top: a majority of HBV-HDV coinfections lead to the eradication of both viruses. Center: A small portion of HDV superinfections of HBsAg-positive patients are self-limited. Bottom: Most HDV superinfections of HBV patients and a minority of HBV-HDV coinfections lead to chronic HDV infection. ALT: alanine transaminase; Ig: immunoglobulin. Created with BioRender.com.

Two distinct infection modes have been identified: HBV-HDV coinfection and HDV superinfection (Negro, 2014). Coinfection involves the simultaneous infection of both viruses, resulting in acute hepatitis with an incubation period of 8–12 weeks. This can manifest as mild to severe or even fulminant hepatitis, with symptoms that can be challenging to differentiate from other forms of acute viral hepatitis. These symptoms typically appear a few weeks after infection and include fatigue, loss of appetite, nausea, vomiting, dark urine, pale stools, and jaundice. Severe liver damage can lead to a high mortality rate. Over 95% of coinfected patients can successfully clear both viruses, while less than 5% develop chronic hepatitis D (Farci and Niro, 2012). HDV RNA and HDAg serve as early and sensitive markers but disappear quickly. However, anti-HDV IgM is not a specific marker for coinfection, and IgG levels start lower and appear later. These are the only detectable markers within 6–12 months after infection in patients with noticeable symptoms, but diagnostic confirmation may be missed if patients with mild symptoms are not tested or are tested too late (see Figure 2).

In contrast to HBV mono-infection and HBV-HDV coinfection, HDV superinfection leads to the most severe forms of viral hepatitis (Miao et al., 2020; Romeo et al., 2009; Manesis et al., 2013; Buti et al., 2011). Superinfection refers to HDV infection on the basis of chronic HBV infection (Negro and Lok, 2023). It can accelerate cirrhosis development within an average of 5 years and significantly increases the risk of liver cancer within 10 years (Stockdale et al., 2020). HDV can be cleared in 20% of superinfected patients, but it persists in up to 80% of cases (Stockdale et al., 2020; Shen et al., 2020; Buti et al., 2021). HDV chronicity typically suppresses pre-existing HBV markers like HBV DNA and HBsAg within a few weeks. Meanwhile, serum HDV RNA levels surge to very high levels. HDAg expression is transient and forms complexes with anti-HDV later on. Both anti-HDV IgM and IgG are positive and persistent (see Figure 2). Regardless of coinfection or superinfection, it is crucial to determine HDV markers and anti-HDV as early as possible. Early diagnosis and treatment can help prevent the progression of chronic hepatitis D and its associated complications.

## 3 HDV clinical screening strategies

Implementation of HDV detection in clinical practice requires a strategic approach due to varying disease burdens and diagnostic capabilities across different regions. Current guidelines from major liver study associations such as the Asia pacific association for the study of the liver (APASL), the European association for the study of the liver (EASL), and the American association for the study of liver diseases (AASLD) recommend universal screening of all HBsAg-positive individuals in various regions (Rizzetto et al., 1977; Sarin et al., 2016; Terrault et al., 2018). This is because HDV infection is aggressive and lacks specific clinical symptoms to distinguish it from HBV monoinfection, making it difficult to identify HDV infections based solely on clinical presentation.

Several HDV screening strategies have been tested and validated. One common approach involves using enzyme-linked immunosorbent assay (ELISA) or chemiluminescence immunoassay (CLIA) for the initial serological testing of anti-HDV antibodies (Sarin et al., 2016; Lin et al., 2020; Rocco et al., 2019). This is typically followed by confirmatory HDV RNA testing using quantitative real-time PCR (qRT-PCR) or droplet digital PCR (ddPCR) to confirm active viral replication and to differentiate between chronic, acute infections, and cured diseases (Yamashiro et al., 2004; Olivero et al., 2022; Xu et al., 2022). This two-step strategy helps reduce the number of false-positive results and ensures that only individuals with true HDV infection undergo further testing and treatment.

Geographically, screening strategies need to be adapted to both high-prevalence and low-prevalence areas. In high-prevalence regions, such as parts of Africa, the Amazon Basin, Eastern Europe, the Mediterranean, the Middle East, and Asia (Chen et al., 2019), widespread screening of all HBsAg-positive individuals is strongly recommended. Early detection can prevent disease progression and reduce the burden of chronic liver disease. In low-prevalence regions, the decision to implement HDV screening depends on several factors, including the availability of testing resources, the cost-effectiveness of screening, and the specific population demographics. Although the overall prevalence of HDV may be lower in these regions, certain high-risk groups within these populations should still be considered for screening. For instance, individuals with a history of intravenous drug use, unprotected sexual contact with individuals from high-prevalence areas, or those who have received blood transfusions or medical procedures in regions with inadequate infection control should be tested for HDV (Niro et al., 1999; Stockdale et al., 2020).

Reflex HDV testing for all HBsAg-positive patients is a strategy that has been proposed and evaluated. This involves automatically performing HDV testing on all samples that test positive for HBsAg without requiring a separate physician order. Reflex testing can help ensure that HDV infections are not overlooked in patients who may not otherwise be tested based on clinical symptoms or risk factors. Studies indicate that this approach can improve HDV detection rates and ensure timely care for patients (Sarin et al., 2016; Terrault et al., 2018). However, the cost-effectiveness and feasibility of reflex testing may vary depending on the local epidemiology of HDV and the availability of testing resources.

In conclusion, the choice of HDV screening strategy should be based on a comprehensive consideration of factors such as disease prevalence, resource availability, and the specific characteristics of the population being screened. Further research and validation are needed to optimize HDV screening strategies and ensure their effective implementation in different clinical settings.

## 4 Diagnostic methods

### 4.1 Anti-HDV

The initial diagnosis of HDV infection relies on the detection of anti-HDV, including serum IgM and IgG. However, a positive result cannot distinguish between a current infection and a past cleared infection. Therefore, active HDV replication is further confirmed through an HDV RNA test. Guidelines from organizations like APASL and EASL recommend an anti-HDV test for individuals who are HBsAg-positive (Sarin et al., 2016; European Association for the Study of the Liver, 2017). AASLD suggests HDV tests not only for HBsAg-positive patients with recent lower or undetectable HBV DNA levels but higher ALT or AST levels but also for individuals at high risk of HDV infection (Terrault et al., 2018). Here are the laboratory diagnostic methods.

#### 4.1.1 Radioimmunoassay

Introduced in the 1980s and 1990s, competitive and non-competitive RIAs were employed to detect anti-HDV antibodies using radioisotopes as markers to label antigens (Rizzetto et al., 1979; Gowans et al., 1990). The Abbott Laboratories anti-HDV RIA kit gained wide use (Bezeaud et al., 1989; Govindarajan et al., 1991; Shattock and Morris, 1991). This method relies on radioactivity intensity for detection, making it highly sensitive. However, it necessitates specialized facilities, involves a time-consuming protocol, and uses hazardous isotopes. RIA was subsequently replaced by an enzyme-linked immunosorbent assay (ELISA) with similar specificity and comparable sensitivity (Kuo et al., 2012).

#### 4.1.2 Enzyme-linked immunosorbent assay

ELISA stands as the most commonly used method for antibody detection (Lin et al., 2020). These kits can detect specific anti-HDV IgG, specific anti-HDV IgM, and total anti-HDV. Anti-HDV IgG constitutes the majority of total antibodies and is present in all patients with a normal immune response to HDV infection (Smedile et al., 1994; Negro and Rizzetto, 1995; Roingeard et al., 1992). The competitive ELISA (e.g., Dia. Pro Diagnostic Bioprobes Srl HDV-Ab ELISA) can detect anti-HDV IgG by binding it to HDAg adsorbed onto the plate. After washing, a peroxidase-conjugated anti-HDV antibody is added, binding to free HDAg. The amount of bound enzyme becomes inversely proportional to the anti-HDV IgG concentration in the tested sample. It’s important to note that the anti-HDV IgM level is low in the late phase of acute HDV infection, and its non-specific binding to HDAg has minimal interference with anti-HDV IgG detection. Total anti-HDV are a crucial marker and can be detected using a competitive ELISA (e.g., DiaSorin ETI-AB-DELTAK-2).

Anti-HDV IgM typically becomes detectable 2–3 weeks after the onset of HDV infection symptoms and disappears within 2 months after acute HDV infection (Koh et al., 2019; Wranke et al., 2014; Mederacke et al., 2012). The principle behind the anti-HDV IgM ELISA involves a capture method that specifically detects anti-HDV IgM, excluding IgG detection (e.g., Dia. Pro Diagnostic Bioprobes Srl HDV-IgM ELISA; DiaSorin ETI-DELTA-IGMK-2). A microplate is coated with an anti-human IgM μ-chain antibody, capturing all IgM antibodies in the sample. Specific HDAg and peroxidase-conjugated anti-HDV Fab antibody are subsequently added for signal development, enabling more accurate detection of anti-HDV IgM compared to other methods (Mederacke et al., 2012).

Previous studies have demonstrated that RIA and ELISA exhibit similar sensitivity for detecting low-titer IgG antibodies. However, in the case of high-titer IgG samples, ELISA reaches the endpoint faster than RIA (Govindarajan et al., 1991; Kuo et al., 2012). ELISA is a straightforward and safer method compared to RIA, with practical advantages. Nevertheless, it can only be utilized for serum/plasma samples, and the assay is qualitative since most commercial kits do not provide anti-HDV standard samples.

#### 4.1.3 Chemiluminescence immunoassay (CLIA)

CLIA technology utilizes magnetic particles coated with recombinant HDAg to capture anti-HDV IgM and IgG from the samples. A monoclonal antibody targeting human IgM/IgG, labeled with luminol, isoluminol, or other luminescent substrates, is employed for signal development. The emitted photons are then detected by chemiluminescence detectors (e.g., Dia. Pro Diagnostic Bioprobes Srl Sara/CLIA; DiaSorin LIAISON XL Murex). CLIA offers several advantages, including higher sensitivity compared to ELISA and a shorter assay time. Notably, DiaSorin S. p.A. developed an automated anti-HDV CLIA, LIAISON XL Murex, which provides random access to samples and delivers results in as little as 32 min. Rocco et al. conducted a comparison between LIAISON and the ETI-AB-DELTAK-2 ELISA for total anti-HDV (Rocco et al., 2019), revealing that CLIA had a lower detection limit and reasonable concordance with ELISA. However, it remains a qualitative method and requires specialized laboratory equipment.

#### 4.1.4 Quantitative microarray antibody capture

Chen et al. devised a high-throughput Q-MAC assay, involving the microarray printing of recombinant S-HDAg on slides coated with a plasmonic gold film. Diluted serum samples were applied to each well, followed by the addition of IRDye800-labeled anti-human IgG secondary antibodies. The fluorescent signal was then detected using a Licor Odyssey scanner. Purified anti-HDV IgG from patient sera served as an internal standard (Chen et al., 2017). An important aspect of this assay is its ability to provide quantitative and defined anti-HDV titers, which can be used to predict HDV RNA positivity prospectively (Mahale et al., 2018; Mahale et al., 2019).

#### 4.1.5 Lateral flow assay

LFA is a point-of-care test that does not require a laboratory instrument. It involves immobilizing an engineered pan-genotypic L-HDAg and anti-goat antibody (for detection and control bands, respectively) on a membrane. A filter paper strip with colloidal gold-conjugated anti-human IgG is placed in front of the membrane. In comparison to the DiaSorin ELISA, LFA exhibited a 94.6% sensitivity for ELISA-positive samples and 100% specificity for ELISA-negative samples (Lempp et al., 2021). Although the developer did not test only genotype 4, it showed high specificity and concentration dependence when performing the other genotypes. Expanded access is warranted in regions such as Africa, Asia, and South America, where almost all HDV genotypes are prevalent. And Roggenbach et al. conducted a HDV seroprevalence study in China, utilizing LFA technology on over 4,000 HBsAg-positive sera (Roggenbach et al., 2021).

#### 4.1.6 Immunochromatographic test

Lopes et al. (2025) developed a new method for hepatitis D detection based on the recombinant antigen DTH10.1. The method consists of a DTH10.1 ELISA, an ICT for anti-HDV IgG and a multiplex ICT. The DTH10.1 antigen is based on the bioinformatics analysis of eight HDV genotypes designed to contain a common sequence of HDV antigen. The core of the working principle of the multiplex ICT is the use of colloidal gold nanoparticle labeling technology: DTH10.1 recombinant antigen (covering the conserved sequences of the 8 HDV genotypes) is conjugated to colloidal gold in a 1:1 ratio with a hepatitis B monoclonal antibody (J), which serves as the detection reagent; two lines of detection are set up on a nitrocellulose membrane to immobilize an anti-HBs monoclonal antibody (C, capturing HBsAg) and an anti-human IgG antibody (to capture anti-HDV IgG). When 50 μL of serum sample is added to the assay, HBsAg, if present, binds to colloidal gold-labeled antibody J and migrates to line 1 where it is captured by antibody C to show a red band; anti-HDV IgG binds to colloidal gold-labeled DTH10.1 and is captured by the anti-human IgG antibody in line 2, and the result can be read by the naked eye within 20 min.

These methods are highly sensitive for the detection of different genotypes of hepatitis D virus, ELISA is stable, and the multiplex ICT is suitable for use in remote endemic areas, with analytical sensitivities of up to 5 IU/mL for HBsAg and 95.2% sensitivity and 98.0% specificity for anti-HDV IgG. The ICT is easy to transport and manipulate and provides rapid results. Given the global distribution and genetic variability of HDV, the test should demonstrate high sensitivity to detect infection with all eight HDV genotypes. Moreover, compared to other HBsAg tests, the multiplex ICT detects both markers simultaneously, which is an advantage in endemic areas and provides a more efficient and practical tool for hepatitis D diagnosis.

### 4.2 HDAg

HDAg serves as one of the diagnostic markers for HDV infection, with its detection in the liver and HDV RNA in the blood providing reliable evidence of active HDV replication. Serum HDAg is transiently detectable during the acute phase of HDV infection but forms antigen-antibody complexes with anti-HDV *in vivo*. As a result, the usefulness of HDAg detection is limited and is often effective primarily in primary infections or in immunocompromised patients unable to produce sufficient anti-HDV (Majeed et al., 2023).

#### 4.2.1 Immunohistochemistry/immunofluorescence

Histology remains the gold standard for the most accurate characterization of liver disease and also allows for the classification grading and staging of necroinflammation and fibrosis, respectively (European Association for Study of LiverAsociacion Latinoamericana para el Estudio del Higado, 2015; European Association for the Study of the Liver, 2021). IHC/IF is employed to detect intrahepatic HDAg in liver biopsies. This technique is typically used after serological and other diagnostics have been performed before surgery, allowing IHC/IF to confirm the initial diagnosis. The HDV genotype can be characterized through an IHC assay when genotype-specific anti-HDV are applied (Hsu et al., 2000). IHC/IF is helpful in estimating the prevalence of HDV infection,but unfortunately these additional tests are not available in most pathology laboratories (Olivero and Smedile, 2012).

#### 4.2.2 Immunoblot

Immunoblotting for the detection of serum HDAg is an effective, sensitive, and noninvasive method for diagnosing and monitoring chronic HDV infection (Buti et al., 1989; Jardi et al., 1994). Bergmann, Negro, and Gerin were the first to develop an immunoblot assay for detecting HDAg in the serum and liver of patients as well as acutely infected chimpanzees and woodchucks (Bergmann and Gerin, 1986; Negro et al., 1988). An HDAg-specific antibody is used to detect both S-HDAg and L-HDAg, and a secondary antibody amplifies the signal, enabling its visualization.

#### 4.2.3 ELISA

The early characterization of monoclonal anti-HDV paved the way for the development of HDAg ELISA (Pohl et al., 1987). In this assay, samples are initially incubated with a detergent to dissolve HDAg from HDV particles. The freed HDAg is then captured by pre-coated anti-HDV and detected using a peroxidase-conjugated anti-HDV antibody (e.g., Dia. Pro Diagnostic Bioprobes Srl HDV-Ag ELISA). Despite its sensitivity, HDAg levels decline and become undetectable over years in chronic infections compared to HDV RNA (Negro et al., 1988).

### 4.3 HDV RNA

While anti-HDV IgM can be detected during acute and chronic infections in the acute phase, and the presence of anti-HDV IgG cannot differentiate between chronic and resolved acute infections (see Figure 2), further assessment of HDV RNA is necessary. HDV RNA serves as the most direct evidence of active HDV replication and is considered the gold standard for diagnosis. The efficacy of antiviral therapy also relies on accurate HDV RNA evaluation (Terrault et al., 2018; Wedemeyer and Manns, 2010).

#### 4.3.1 Northern blot and slot blot

Serum HDV RNA was probed using Northern blot hybridization, employing a radiolabeled cloned cDNA fragment or its RNA transcript (Denniston et al., 1986; Smedile et al., 1986). The Northern blot involves serum RNA extraction, denaturation, gel electrophoresis separation, transfer to cellulose nitrate or nylon membrane, hybridization with radiolabeled HDV DNA or transcripts, and signal development. The use of a riboprobe is more sensitive than a homologous DNA probe (Smedile et al., 1990). This assay is semi-quantitative, non-invasive, and the only way to visualize HDV RNA dimers and trimers. Slot blot simplifies the process by bypassing RNA fractionation and transfer steps, making it more straightforward than Northern blot (Taylor et al., 1987; Saldanha et al., 1989). Both blots have low throughput and lower sensitivity compared to PCR, and as a result, they have been largely replaced by PCR technology.

#### 4.3.2 Fluorescence in situ hybridization

FISH utilizing a nonradioisotopic strand-specific HDV probe is a rapid and sensitive method for detecting HDV RNA genomes in paraffin-embedded liver biopsies. In certain cases, hepatic HDV RNA may be the only positive marker, while HDAg and serum HDV RNA are undetectable (Lopez-Talavera et al., 1993; Negro, 2004). When combined with confocal microscopy, this method (e.g., Advanced Cell Diagnostics RNAscope) enables the visualization of HDV RNAs in hepatocyte nuclei and quantification of genomic/antigenomic copy numbers (Gowans et al., 1988; Cunha et al., 1998).

#### 4.3.3 Reverse transcription (RT-)PCR

In comparison to hybridization methods, PCR amplification of reverse-transcribed HDV RNA offers several advantages in terms of sensitivity, simplicity, and clinical feasibility (Madejón et al., 1990; Zignego et al., 1990). Madejón et al. demonstrated that RT-PCR is 10,000 times more sensitive than slot blot (Madejón et al., 1990). Serum HDV RNA is reverse transcribed, and the resulting complementary (c)DNA product serves as the template for exponential amplification using a primer pair that selectively binds to conserved sequences, such as the HDV ribozyme. RT-PCR products not only reveal HDV RNA levels but can also be utilized for HDV genotyping and cloning (Roggenbach et al., 2021). Moreover, the sensitivity and specificity of RT-PCR have been enhanced. Nested RT-PCR employs an external primer pair along with an internal pair that amplifies the first-round PCR product, making it suitable for detecting RNA with lower copy numbers (Roggenbach et al., 2021; Salichos et al., 2023). Thus, while RT-PCR has a detection limit of 1,000 genome copies/mL, nested RT-PCR can detect as few as 10 genome copies/mL (Smedile et al., 2004). Currently, the use of reverse transcription-nested PCR (RT-nested PCR) for genotyping HDV has become the gold standard in the diagnosis of occult hepatitis B infection (Ceesay et al., 2022). A 2016 study employed a semi-nested RT-nested PCR method to detect HDV isolates from blood donors in Sudan who were coinfected with HBV/HDV. This approach has been demonstrated to be both sensitive and specific for HDV/HBV co-infection in this population (Mohmed et al., 2016).

#### 4.3.4 Quantitative real-time (qRT-)PCR

In contrast to RT-PCR, qRT-PCR is quantitative and real-time, relying on the detection of DNA-bound fluorescent reporters (e.g., SYBR-Green dye, Taqman probe, FRET probe, and molecular beacon). Yamashiro et al. were the first to use qRT-PCR to detect serum HDV RNA in 48 patients with positive HBsAg and anti-HDV, establishing a correlation between HDV RNA loads and disease stages (Yamashiro et al., 2004). Le Gal et al. further demonstrated that viral loads reflect profiles of virological responses during interferon therapy (Le Gal et al., 2005). Combining results from anti-HDV and HDV RNA via qRT-PCR (e.g., RoboGene HDV RNA kit) can differentiate between chronic and resolved infections (Castelnau et al., 2006). However, the vast inter-laboratory variability in diagnostic qRT-PCR assays, which employ different standards, primers, and protocols, necessitates international standardization for this widely-used method (Le Gal et al., 2016).

#### 4.3.5 Droplet digital (dd)PCR

The third-generation PCR technology includes chip and droplet dPCR, which are highly sensitive, extremely accurate, and not dependent on standard samples. ddPCR employs microfluidic technology to divide one sample into 20,000 water-in-oil emulsified micro-droplets, with most containing a few nucleic acid templates for independent qPCR amplification and fluorescence detection. Absolute quantification of copy numbers is based on the events of positive and negative fluorescent droplets conforming to a Poisson distribution, eliminating the need for an external standard curve. This method operates within a quantitative dynamic range of 10^1^–10^6^ IU/mL, with a lower limit of quantification at 8.76 IU/mL (Olivero et al., 2022; Xu et al., 2022). ddPCR is highly specific and capable of detecting exceptionally low levels of HDV, surpassing the accuracy of qRT-PCR (Xu et al., 2022; Hindson et al., 2013). Furthermore, a duplex ddPCR allows for the quantification of both non-edited and ADAR1-edited HDV genomes in a single sample following FTI-277 treatment (Verrier et al., 2022). Serological assays were validated to quantify HDV RNA editing rates, enabling comprehensive investigation of their correlation with disease progression patterns and therapeutic outcomes in chronic hepatitis D patients. It’s worth noting that this method is complex, expensive, and not widely adopted. Despite its advantages, ddPCR is currently limited by its complexity and high cost, restricting its routine clinical application. It remains primarily a research tool and has not yet been widely adopted in clinical settings due to these practical constraints.

#### 4.3.6 Reverse transcription-loop-mediated isothermal amplification

RT-LAMP is a diagnostic assay that involves continuous cycling of DNA synthesis using inner and outer primers and Bst polymerase at temperatures ranging from 60°C–65°C (Notomi et al., 2000). Wang et al. developed an RT-LAMP system for HDV detection, requiring only a 50-min reaction at 65°C, with a lower detection limit of 75 fg/μL (Wang et al., 2013). This method is rapid, straightforward, 1,000 times more sensitive than PCR, and does not necessitate large instruments. However, it has thus far been applied to only a limited number of genotype 1 samples, requiring further optimization for broader patient sample and genotype detection. While RT-LAMP offers a potential solution for point-of-care testing with its simplicity and speed, it requires further optimization to cover a broader range of HDV genotypes. As of now, its application has been limited to specific genotypes, and it is not yet a fully validated clinical diagnostic method.

#### 4.3.7 Next-generation sequencing

The HDV quasispecies that accumulate during prolonged HDV replication can be characterized using NGS (Beeharry et al., 2014; Homs et al., 2016). In comparison to Sanger sequencing, NGS offers more precise quantification of edited and unedited HDV genomes, as well as other mutations in patient serum samples (Sopena et al., 2018). Additionally, NGS enables rapid and accurate HDV genotyping (Sopena et al., 2018; Wu et al., 2020). Depending on the HDV genotype and strain, variable RNA editing rates in patients have been observed (Dziri et al., 2021). NGS can also analyze the evolution of ribozyme quasispecies (QS) and identify highly conserved regions as potential gene - silencing targets (Pacin-Ruiz et al., 2022). Owing to the varying replication capacities of HDV genotypes and the distinct impacts of the HBV envelope on viral particle assembly and infectivity, coupled with the clinical symptoms induced by different HDV genotypes differing, the employment of genetic sequencing to precisely delineate genotypes is particularly crucial. Furthermore, this is of significant importance for the compilation of regional prevalence rates of HDV. NGS, though powerful in providing detailed insights into HDV quasispecies and genotyping, is currently hindered by high costs and technical complexity. These factors limit its routine use in clinical laboratories, confining it mainly to research settings where resources and expertise are more readily available.

## 5 Challenges and perspectives

There are several challenges in current HDV diagnostic approaches. Firstly, most diagnostic anti-HDV methods are qualitative. Having absolute IgM/IgG levels could help evaluate disease severity and treatment effectiveness. The absence of an antibody standard makes it difficult to compare specificity and sensitivity between different methods (Table 1). Several comparative studies have demonstrated significant differences in detection sensitivity among different anti-HDV detection reagents in commercially available products (Bezeaud et al., 1989; Shattock and Morris, 1991; Kuo et al., 2012; Chow et al., 2016). Therefore, establishing anti-HDV standards is essential for comparing experimental data from various laboratories, kits, and methods.

**TABLE 1:** Diagnostic methods for hepatitis D.

Assay	Target	SC	SS	Significance	Use	Comments	Refs
RIA	IgM; IgG	+++	++	Radioisotopes are markers	Mainly for research purposes	Products not provided on the market	Rizzetto et al. (1979), Govindarajan et al. (1991)
ELISA-Ab	IgM; IgG; total	+++	++	Most commonly used; qualitative	Clinical and research	Standard samples are not provided	Lin et al. (2020), Wranke et al. (2014), Mederacke et al. (2012)
CLIA	IgM; IgG; total	+++	+++	Automation and high-throughput	Clinical and research	A special instrument is required	Rocco et al. (2019)
Q-MAC	IgG	+++	++	High throughput; qualitative	Mainly for research purposes	Point-of-care if further optimized	Chen et al. (2017), Mahale et al. (2018), Mahale et al. (2019)
LFA	IgG	+++	++	Simple; qualitative; point-of-care	Mainly for research purposes	Lower sensitivity than ELISA	Lempp et al. (2021), Roggenbach et al. (2021)
ICT	IgG	++++	+++	Fast; Qualitative, detecting HBsAg	Mainly for research purposes	Cross-reactivity present; preliminary screening	Lopes et al. (2025)
IHC/IF	Liver HDAg	++	++	Precise HDAg tissue localization; single cell	Mainly for research purposes	Invasive; not for primary screening	Hsu et al. (2000)
IB	S/L-HDAg	++	+	Differentiation with two HDAgs	Mainly for research purposes	A large amount of starting material	Bergmann and Gerin (1986), Negro et al. (1988)
ELISA-Ag	Serum HDAg	++	+++	More sensitive than IB	Mainly for research purposes	Serum HDAg is not a reliable marker	Pohl et al. (1987)
NB and SB	Serum HDV RNA	++	+	Low throughput; detection of HDV multimers	Mainly for research purposes	Much lower sensitivity than PCR	Smedile et al. (1986), Smedile et al. (1990)
FISH	Liver HDV RNA	+	+++	Precise RNA tissue localization; single cell	Mainly for research purposes	Invasive; unspecific signals	Lopez-Talavera et al. (1993), Gowans et al. (1988)
RT-PCR	Serum HDV RNA	++	+/++(#)	Less sensitive and specific	Mainly for research purposes	Used for genotyping and cloning	Madejón et al. (1990), Smedile et al. (2004)
qRT-PCR	Serum HDV RNA	+++	++	Most commonly used; quantitative	Clinical and research	Inter-laboratory variability; standards are heterogenous	Yamashiro et al. (2004), Le Gal et al. (2005), Le Gal et al. (2016)
ddPCR	Serum HDV RNA	+++	+++	Standards not required; lower LOQ	Mainly for research purposes	Detection of unedited and edited RNA	Olivero et al. (2022), Xu et al. (2022), Verrier et al. (2022)
RT-LAMP	Serum HDV RNA	+++	+++	Fast and sensitive	Mainly for research purposes	Point-of-care if further optimized	Wang et al. (2013)
NGS	Serum HDV RNA	+++	+++	Detection of HDV quasispecies	Mainly for research purposes	Relatively higher cost	Homs et al. (2016), Sopena et al. (2018), Dziri et al. (2021)

RIA, radioimmunoassay; ELISA, enzyme-linked immunosorbent assay; CLIA, chemiluminescence immunoassay; Q-MAC, quantitative microarray antibody capture; LFA, lateral flow assay; IHC/IF, immunohistochemistry/immunofluorescence; IB, immunoblot; NB, Northern blot; SB, slot blot; FISH, fluorescence *in situ* hybridization; RT-PCR, reverse transcription PCR; qRT-PCR, quantitative real-time PCR; ddPCR, droplet digital PCR; RT-LAMP, reverse transcription-loop-mediated isothermal amplification; NGS, next-generation sequencing; Ag, antigen; Ab, antibody; SC, specificity; SS, sensitivity. +++, very high; ++, high; +, medium. #, for nested RT-PCR., Use:use cases.

Secondly, as previously mentioned, genomes and HDAg among the eight genotypes exhibit high heterogeneity. Most immunological methods use recombinant antigen/antibody from a specific HDV1 genotype (Gowans et al., 1990; Lu et al., 2016). This may result in reduced detection sensitivity for other genotypes like HDV3 and HDV5-8 found in remote regions. Considering factors such as the geographical differences of HDV strains, the known genotypes and related limitations of the main relevant detection techniques are summarized in (Table 2). In the mentioned LFA, a recombinant L-HDAg was used from an alignment that included 54 HDAg sequences from all genotypes, but HDV4 was not determined (Lempp et al., 2021). Cross-genotype comparisons are still necessary for most assays. Besides genotypes, the HDV genome has a high GC content and extensive secondary structure (Wang et al., 1986). Protocols with varying RNA denaturation and primer binding conditions have shown significant variability (Le Gal et al., 2016). The first WHO international HDV RNA standard, established in 2013 from an HDV1 patient serum, contained 575,000 IU/mL. However, the published sequence and the one determined in the actual standard were not identical (Pyne et al., 2017). The WHO IS is available from the Paul-Ehrlich-Institut, Langen, Germany (www.pei.de).

**TABLE 2:** Known genotypes of detection technology and their related limitations.

Assay	Genotypic	Limitations	Refs
RIA	—	The process is time-consuming and involves the use of dangerous isotopes	Rizzetto et al. (1979), Govindarajan et al. (1991)
ELISA-Ab	Mainly targeting HDV-1	Insufficient sensitivity to HDV types 5–8, HDV type 3 and HDV type 4. Unavoidable missed detection	Lin et al. (2020)
CLIA	—	Unable to distinguish genotype or active infection	Rocco et al. (2019)
Q-MAC	HDV1-8	It is not explicitly stated whether the clinical samples of all genotypes have been experimentally verified, relying on specific equipment and reagents	Chen et al. (2017), Mahale et al. (2019)
LFA	HDV1-3,5-8	Undetected HDV type 4 samples, Insufficient sensitivity for samples with low titers and incomplete coverage of some genotypes	Lempp et al. (2021), Roggenbach et al. (2021)
ICT	HDV1-8	Compared with other genotypes, the detection sensitivity of HDV-5 and HDV-8 is low, and there is cross-reactivity	Lopes et al. (2025)
IHC	HDV1-2	Low sensitivity, standardized kits have not yet been widely used	Hsu et al. (2000)
IB	—	The detection effect is limited by the design of the antibody and the form of the antigen present in the sample	Jardi et al. (1994), Bergmann and Gerin (1986)
ELISA-Ag	—	Insufficient genotype coverage	Pohl et al. (1987)
Northern/Slot blot	HDV1-8	Low flux and low sensitivity,semi-quantitative	Smedile et al. (1990), Chen et al. (1986)
FISH	—	Multiple detections are restricted and sample requirements are stringent	Lopez-Talavera et al. (1993), Negro, 2004, Cunha et al. (1998)
RT-PCR	HDV1-8	For highly variable genotypes (such as HDV-5–8), it may be necessary to optimize primers or combine multiplex PCR.	Roggenbach et al. (2021), Ceesay et al. (2022)
qRT-PCR	HDV1-8	Pay attention to disease relevance, and there is no unified standard	Yamashiro et al. (2004), Le Gal et al. (2005), Le Gal et al. (2016)
ddPCR	HDV1-8	Genotype detection is limited by fluorescence channels, and the reagents are expensive	Olivero et al. (2022), Verrier et al. (2022)
RT-LAMP	HDV1	Impossible to accurately classify, and other genotypes are not currently covered	Notomi et al. (2000), Wang et al. (2013)
NGS	HDV1-8	The universality of the probe is insufficient	Sopena et al. (2018), Wu et al. (2020)

RIA, radioimmunoassay; ELISA, enzyme-linked immunosorbent assay; CLIA, chemiluminescence immunoassay; Q-MAC, quantitative microarray antibody capture; LFA, lateral flow assay; IHC/IF, immunohistochemistry/immunofluorescence; IB, immunoblot; NB, Northern blot; SB, slot blot; FISH, fluorescence *in situ* hybridization; RT-PCR, reverse transcription PCR; qRT-PCR, quantitative real-time PCR; ddPCR, droplet digital PCR; RT-LAMP, reverse transcription-loop-mediated isothermal amplification; NGS, next-generation sequencing.

Thirdly, many assays are manual and have low throughput. The application of highly automated diagnostic methods offers the advantages of reliability, reproducibility, and the ability to scale tests dynamically compared to manual workflows. Pfluger et al. developed a new quantitative HDV qRT-PCR assay on the Roche COBAS6800 system (Pflüger et al., 2021). This assay encompasses all eight genotypes and achieves a lower limit of quantification of 10 IU/mL.

Fourthly, there is a need for HDV screening in countries where information on HDV prevalence is lacking. Given the limitations of large instruments and the availability of qualified technicians in remote areas, the development of affordable and simple point-of-care tests is a future goal. In this regard, Q-MAC, LFA, and RT-LAMP assays show promise for further optimization.

In conclusion, it is essential to establish international HDV standards, develop pan-genotypic primers, and standardize protocols for existing diagnostic methods, as well as create novel point-of-care tests. These efforts are crucial for reducing false negatives, improving specificity and sensitivity in the mentioned assays, and enabling early and precise diagnosis, which can lead to timely treatment and potentially prevent HDV-related liver damage, thus reducing the medical burden. Moreover, some technologies, due to their high cost, low throughput, or high requirements for equipment or technicians, are currently mainly used as research tools. After further optimization and validation, these technologies may play a greater role in clinical diagnosis in the future.
