# Correction: A comprehensive review of diagnostic approaches for hepatitis D

**DOI:** 10.3389/fmolb.2026.1842937

**Published:** 2026-04-17

**Authors:** Frontiers Editorial Office

**Affiliations:** Frontiers Media SA, Lausanne, Switzerland

**Keywords:** hepatitis D, HDV, HDV RNA, HDAg, anti-HDV, diagnosis, screening

With this notice, Frontiers states its awareness of misrepresentation of the availability of the 1st World Health Organization (WHO) International Standard (IS) for HDV RNA for nucleic acid amplification technique assays (code number 7657/12) in this article. The WHO IS is available from the Paul-Ehrlich-Institut, Langen, Germany (www.pei.de).

A correction has been made to Section **5 Challenges and perspectives**, second paragraph. The original article has been updated. 

